# Effectiveness of pharmacological therapies for fibromyalgia syndrome in adults: an overview of Cochrane Reviews

**DOI:** 10.1093/rheumatology/keae707

**Published:** 2024-12-20

**Authors:** Andrew Moore, Julia Bidonde, Emma Fisher, Winfried Häuser, Rae Frances Bell, Serge Perrot, Souzi Makri, Sebastian Straube

**Affiliations:** Newton Ferrers, Plymouth, UK; School of Rehabilitation Science, University of Saskatchewan, Saskatoon, SK, Canada; Centre for Pain Research, University of Bath, Bath, UK; Department of Psychosomatic Medicine and Psychotherapy, Technische Universität München, München, Germany; Regional Centre of Excellence in Palliative Care, Haukeland University Hospital, Bergen, Norway; Centre de la Douleur, Hôpital Cochin, Université Paris Cité, INSERM U987, Hôpital Cochin, Paris, France; Cyprus League of People with Rheumatism, Nicosia, Cyprus; Division of Preventive Medicine, Department of Medicine, University of Alberta, Edmonton, AB, Canada; School of Public Health, University of Alberta, Edmonton, AB, Canada

**Keywords:** fibromyalgia syndrome, duloxetine, milnacipran, pharmacological interventions, pregabalin, systematic overview, systematic reviews

## Abstract

**Objectives:**

To summarize and evaluate Cochrane reviews of pharmacological therapies for adults with fibromyalgia syndrome (FMS) pain.

**Methods:**

Systematic search of Cochrane Database of Systematic Reviews to May 2024. Generic quality assessment used AMSTAR-2 criteria, validity checks of potentially critical factors in evaluation of analgesic efficacy and assessment of susceptibility of results to publication bias. Pain outcomes were participant-reported pain relief of ≥30% or ≥50%, or PGIC much or very much improved.

**Results:**

Twenty-one reviews (87 trials, 17 631 patients) were included. All rated moderate (15) or high-quality (6) using AMSTAR-2 and at least seven of eight critical pain criteria were met by 13 of 21 reviews. Diagnosis of FMS used recognized criteria. Seven reviews found no trials (carbamazepine, clonazepam, lamotrigine, phenytoin, oxycodone, topiramate or valproate), seven had limited and inadequate data (antipsychotics, cannabinoids, combination therapy, gabapentin, lacosamide, monoamine oxidase inhibitors, NSAIDs) and two were subject to publication bias (amitriptyline, SSRI). Mirtazapine had moderate evidence of no effect. Duloxetine, milnacipran and pregabalin had moderate/good evidence of substantial pain relief for 4–12 weeks in around 1 in 10 adults with moderate or severe FMS pain, without evidence of efficacy beyond six months. Serious adverse events were no more common than with placebo. There was no evidence about who might benefit or experience adverse events. There was no substantial efficacy evidence for other medicines.

**Conclusions:**

Duloxetine, milnacipran and pregabalin had good evidence that about 1 person in 10 with moderate or severe pain experienced pain intensity reduction by at least 50%.

## Graphical abstract

**Figure:**
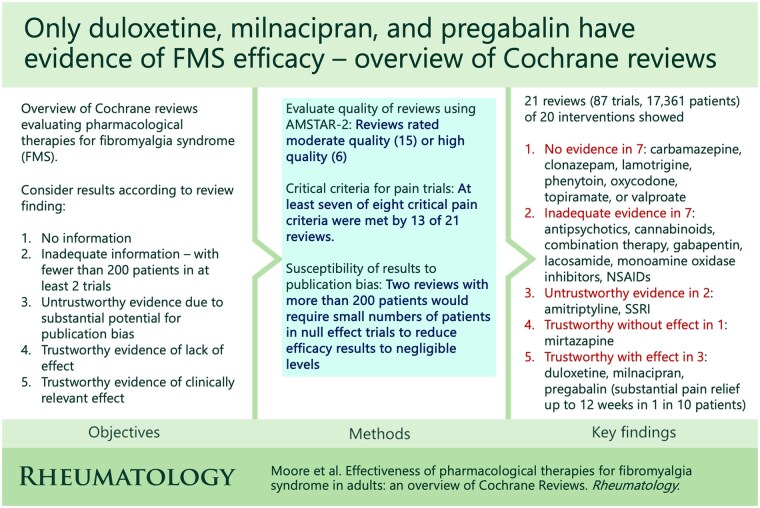

Rheumatology key messages

- Duloxetine, milnacipran and pregabalin showed substantial pain relief in about 1 in 10 adults; pain benefits were not associated with other symptom benefits for these three drugs in the Cochrane Reviews.
- Reviews could not inform which adults might benefit or experience adverse events.
- Most reviews found no trials (carbamazepine, clonazepam, lamotrigine, phenytoin, oxycodone, topiramate or valproate) or only low-quality evidence subject to bias (antipsychotics, cannabinoids, combination therapy, gabapentin, lacosamide, monoamine oxidase inhibitors, NSAIDs, amitriptyline, SSRI).

## Introduction

Cochrane Reviews facilitate overviews of evidence as they are undertaken according to standard guidance and informed by criteria for what constitutes reliable evidence in chronic pain in general [1] and in FMS [2]. This overview of Cochrane reviews of pharmacological interventions for FMS complements another of the efficacy of non-pharmacological interventions [3].

FMS was defined as widespread pain lasting longer than three months, with pain on palpation at 11 or more of 18 specified tender points [4]. The International Classification of Diseases (ICD-11) places FMS under Chronic Widespread Pain (CWP; Code MG30.01). FMS is defined as a form of CWP (pain in at least four of five body regions or in at least three or four body quadrants) associated with sleep disorders, cognitive dysfunction and somatic symptoms present for at least three months and not better accounted for by another diagnosis [5, 6].

Fibromyalgia is common, with a global mean prevalence of 2.7% (range 0.4% to 9.3%) with a mean prevalence of 3.1% in the Americas, 2.5% in Europe and 1.7% in Asia [7]. Fibromyalgia is more common in women, with a female to male ratio of 3:1.

FMS has a heterogeneous clinical presentation, associated with anxiety and depressive disorders and chronic secondary pain syndromes like inflammatory rheumatic diseases and osteoarthritis [8]. Recent guidelines recommend a stepwise graduated approach depending on symptoms and disability [9, 10], starting with education, non-pharmacological therapies and psychological therapies, although there is limited evidence of their effectiveness [3, 11, 12]. An overview of Cochrane reviews of non-pharmacological therapies demonstrated only low certainty evidence for any efficacy, none had any substantial effect, with sparse evidence about adverse events [3].

Pharmacological interventions are recommended as part of a multidisciplinary approach combined with physical and/or cognitive interventions for severe forms of FMS [9, 10]. Treatment is often by antidepressants (typically duloxetine and amitriptyline [13–15]) or antiepileptics (typically gabapentin or pregabalin [16–19]). Substantial (worthwhile) pain relief is achieved by a small proportion of patients [20, 21]. Individuals experiencing substantial levels of pain relief with pregabalin also benefited from substantial improvements in other symptoms, of fatigue, function, sleep, depression, anxiety and ability to work, with significant improvement in quality of life [22, 23]. Good response in only a small proportion of people is typical of chronic pain conditions [24].

Standards used to assess evidence in chronic pain trials pay particular attention to trial duration, withdrawals and statistical imputation following withdrawal, which can substantially alter estimates of efficacy. An important recent change is the move towards assessing the number of participants who experience a large decrease in pain (by at least 50%) continuing in treatment in trials of 8–12 weeks or longer rather than average measures. Pain intensity reduction of 50% or more has been correlated with improvements in comorbid symptoms, function and quality of life for people with chronic pain [20, 25, 26] and FMS [21, 23]. An overview of the evidence for these outcomes is relevant for people with FMS and their carers.

The primary objectives of this overview were to summarize the therapeutic effectiveness and safety of pharmacological therapies for pain compared with placebo in adults with FMS.

## Methods

A protocol for the overview was published [27]. Amendments to the protocol PRIOR statement are in Supplementary Data S1 and Supplementary Data S2 [28], available at *Rheumatology* online.

We included Cochrane reviews of pharmacological therapies in adults (≥18 years) diagnosed with FMS using an established diagnostic criterion [5, 29–35]. Reviews of children or mixed populations not reporting outcomes separately were excluded. Any pharmacological therapy and any comparator were eligible. We sought outcomes as close as possible to three months (13 weeks) after treatment; those earlier than one month (four weeks) after beginning treatment were not eligible.

Eligible study designs were RCTs or non-RCTs providing details of inclusion and exclusion criteria; databases searched and search strategies; patient-reported pain or pain relief; and summary results for at least one desired outcome. We searched the Cochrane Database of Systematic Reviews 2021 issue 12 of Cochrane Library Wiley interface on 1 January 2022, using no limits on date or language, and checked again on 1 May 2024.

Two reviewers independently selected reviews for inclusion, carried out assessments of methodological quality, extracted data, analysed data if required, assessed how the review authors used the GRADE criteria, and made their own GRADE assessments based on the information provided. Reviewers resolved disagreements by consensus among all authors; all authors saw all data.

Information was collected on the number of included studies and participants; medicine, dose and route of administration; baseline demographic characteristics and pain; and any additional methodological information of importance. Outcomes sought are listed in Supplementary Data S3, available at *Rheumatology* online.

Methodological quality was assessed using AMSTAR-2 criteria [36], with additional validity checks of potentially critical factors in the evaluation of analgesic efficacy (Supplementary Data S4, available at *Rheumatology* online) [37].

Susceptibility of results to publication bias was estimated by calculating the number of participants in studies with zero effect [risk ratio (RR)=1] needed to give a number needed to treat to benefit (NNTB) too high to be clinically relevant [38]. Cut-off values for clinical relevance were NNTB values of 10 and 20 for the outcome of participant-reported pain relief of ≥30% or ≥50%.

Analgesic efficacy estimates used placebo as a common comparator as direct comparisons are rare [39, 40]. Any direct comparisons were noted. No further data synthesis was planned, but if indicated, data from at least 200 participants had to be available [41]. We calculated RR or risk difference (RD) with 95% confidence intervals (CIs) using a fixed-effect model [42]. We used or calculated NNTB and number needed to harm (NNTH) with 95% CIs using the pooled number of events [43]. We assumed a statistically significant difference from control when the 95% CI did not include unity for RR or zero for RD.

We made an independent assessment of GRADE to assess the certainty of the evidence [44], and compared this to how the review authors had used GRADE criteria, using the amount and certainty of evidence to report results in a hierarchical way [45, 46]. We split results into five groups, essentially according to the GRADE descriptors.

1. Medicines and doses for which Cochrane reviews found no information (very low-certainty evidence).
2. Medicines and doses for which Cochrane reviews found inadequate information: fewer than 200 participants in comparisons, in at least two studies (very low-certainty evidence in the review).
3. Medicines and doses for which Cochrane reviews found evidence of effect, but where results were potentially subject to publication bias. We considered the number of additional participants needed in studies with zero effect (relative benefit of one) required to change the NNTB for at least 50% maximum pain relief to an unacceptably high level (in this case the arbitrary NNTB of 10) [38]. Where this number is less than 400 (equivalent to four studies with 100 participants per comparison, or 50 participants per group), the results are susceptible to publication bias and therefore unreliable (low-certainty evidence).
4. Medicines and doses for which Cochrane reviews found trustworthy evidence of no effect: more than 200 participants in comparisons, but where there was no statistically significant difference from placebo (moderate- or high-certainty evidence).
5. Medicines and doses for which Cochrane reviews found trustworthy evidence of clinically relevant effect, where results were reliable and not subject to potential publication bias (high-certainty evidence).

## Results

**Figure 1.**
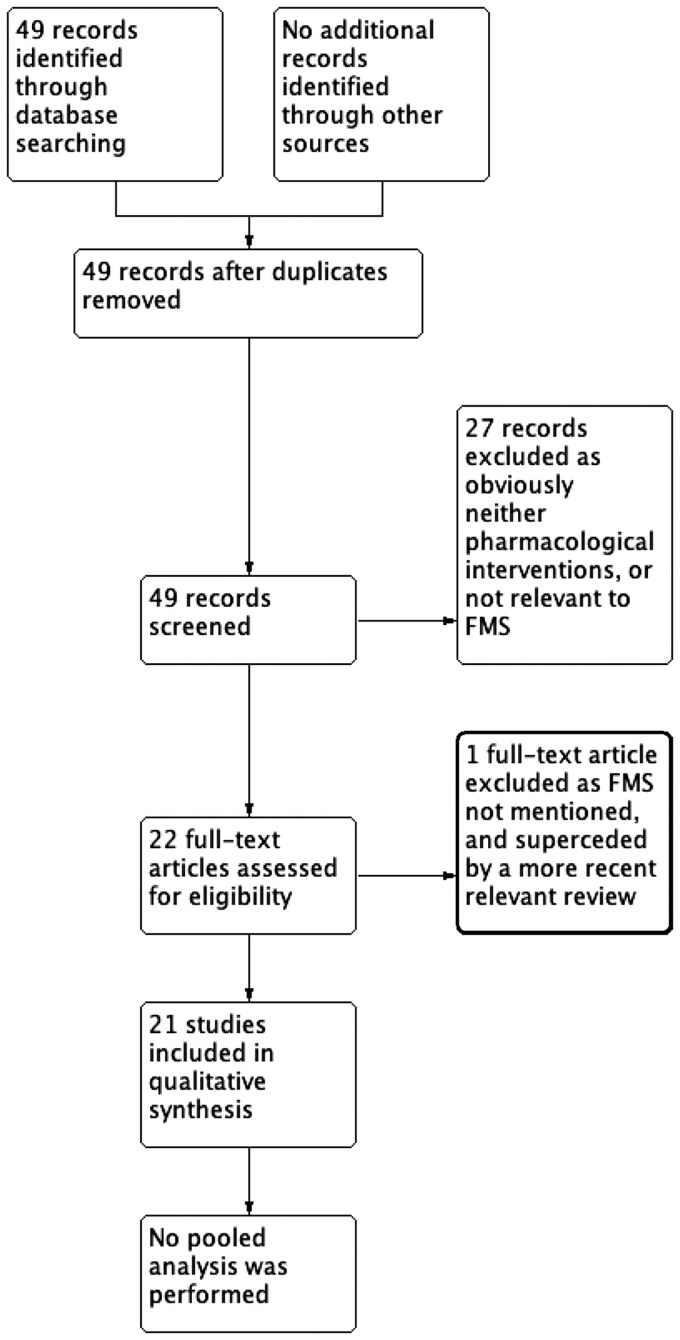
Study flow diagram

Searches identified 49 Cochrane reviews. We excluded 27 reviews at initial screening and one after full text assessment (Fig. 1; Supplementary Data S5, available at *Rheumatology* online); 21 reviews were included. Seven reviews were of antidepressants including selective serotonin reuptake inhibitors (SSRIs) [14, 47–52], nine of antiepileptics [16, 53–60] and one each of antipsychotics [61], cannabinoids [62], oxycodone [63], NSAIDs [64] and combination therapy (details in Supplementary Table S6 and Supplementary Table S7, available at *Rheumatology* online) [65]. Duloxetine [14] and milnacipran [47] were reviewed separately and as part of a review of serotonin and noradrenaline reuptake inhibitors (SNRIs) [52]. We used the most recent data available.

Seven Group 1 reviews found no trials [53, 55, 57–60, 62], seven Group 2 reviews found inadequate amounts of evidence [16, 49, 56, 61–64], two Group 3 reviews had data potentially subject to publication bias [48, 50], one Group 4 review found good evidence of no effect [51] and four Group 5 reviews found trustworthy evidence of clinically relevant effect [14, 47, 52, 54].

All included reviews involved RCTs using placebo comparator. Most reviews were current or with no update planned. Diagnostic criteria were specified in reviews with data, almost uniformly the American College of Rheumatology (ACR) 1990 or 2010 criteria or variants. The mean age of patients in trials was typically 39–53 years, with typically at least 85% female. Reviews specified or accepted participants with initial pain of at least moderate intensity, typically 4 or more on a 0–10 scale.

Confidence in the certainty of the evidence using AMSTAR-2 was high in six reviews and moderate in 15. For seven of the reviews with moderate confidence, scoring was difficult because the reviews had few or no trials or participants, which meant that some items could not be scored. At least seven of eight critical pain criteria were met by 13 of 21 reviews, with only four meeting fewer than five criteria (Supplementary Tables S8 and S9, available at *Rheumatology* online).

Reporting of study settings (such as whether the trials recruited participants from the general population or from specialist centres) and of detailed inclusion and exclusion criteria (for example, what comorbidities might have been disallowed) was patchy. Reporting was more consistent in the five reviews in Groups 4 and 5 that typically had large numbers of participants in predominantly large trials.

The comparisons of the GRADE scores of the authors of the original Cochrane reviews and the authors of this overview are in Supplementary Table S10, available at *Rheumatology* online. Six reviews did not use GRADE, principally because there were no data. These reviews were given a GRADE assessment of very low confidence. GRADE assessments were the same in 11 reviews, rated lower by one level by overview authors in one review (because there were fewer than 200 participants) and raised by one level by overview authors in another review because of a different interpretation of GRADE assessment.

### Effects of interventions

#### Pain

Seven Group 1 reviews found no eligible trials of FMS patients (0 trials, 0 patients) involving carbamazepine [60], clonazepam [48], lamotrigine [59], oxycodone [63], phenytoin [54], topiramate [58] or valproate [56]. Our GRADE assessment was that these reviews offered very low certainty evidence.

Seven Group 2 reviews found trials with inadequate information (36 trials, 4196 patients). Three had fewer than 200 participants in at least two studies: cannabinoids (two trials with 72 participants) [62], gabapentin (one trial with 150 participants) [16] and lacosamide (one trial with 159 participants) [59]. Four included trials with >200 participants, but with inadequate information for analysis: monoamine oxidase inhibitors (MAOI) [50], antipsychotics [61], NSAIDs [64] and combination therapy [65]. Our GRADE assessment was of very low certainty evidence.

For MAOI, data for mean pain were available from two trials (121 participants) of unclear risk of bias [50]. For antipsychotic drugs, two trials of quetiapine 50–300 mg daily in 155 participants showed no difference from placebo for ≥50% pain intensity reduction [61]. Twenty of 82 (24%) participants receiving quetiapine and 8/73 (11%) participants receiving placebo reported pain relief of ≥30% (RD 0.12, 95% CI 0.00–0.23; NNTB 8, 95% CI 5–100). Two trials of NSAIDs (146 participants) found no benefit over placebo for ≥50% pain intensity reduction [64], with none for ≥30% pain intensity reduction in three trials (196 participants). A review of combination analgesics included 14 trials (1289 participants), but the clinical heterogeneity across the studies in terms of the class of agents evaluated, specific combinations used, outcomes reported and doses given, combined with the small size of the studies prevented any useful meta-analysis [65].

Two Group 3 reviews (16 trials, 1032 patients) were potentially subject to publication bias. Our GRADE assessment was of very low certainty evidence.

A review of SSRIs combined data on different drugs and doses from six trials and 343 participants and reported an RD of 0.1 (95% CI 0.01–0.2) and NNT of 10 (95% CI 5–100) for ≥30% pain intensity reduction [51]. A calculation of publication bias susceptibility using NNT thresholds of 10 and 20 showed that 0 and 343 participants, respectively, would be required in null effect trials to alter the results. No single trial was scored as low risk of bias for all items, and most trials scored high or uncertain risk for more than one item.

For amitriptyline 25 mg or 50 mg daily, analysis of four trials (275 participants) produced an RD of 0.24 (95%CI 0.14–0.33) and NNT of 4.1 (95% CI 2.9–6.7) for substantial pain relief (equivalent to ≥50% pain intensity reduction) [49]. A calculation of publication bias susceptibility using NNT thresholds of 10 and 20 showed that 396 and 1038, respectively, participants would be required in null effect trials to alter the results. All included trials had at least one high risk of bias with one or more uncertain risks of bias.

One Group 4 review of mirtazapine 15 mg/day to 45 mg/day results from three trials (591 participants) [52] found no statistical difference between mirtazapine and placebo for ≥50% pain intensity reduction (RD 0.05, 95% CI −0.01–0.12), but a significant difference for ≥30% pain intensity reduction (RD 0.13, 95% CI 0.05–0.21). The original review authors downgraded efficacy evidence to low according to GRADE because of indirectness (triallists excluded participants with inflammatory rheumatic diseases and depressive disorders in >50% of studies) and the risk of publication bias. We considered that because the evidence demonstrated so little efficacy, these factors were unlikely to have produced any positive bias, and we rated the GRADE level as moderate.

Four Group 5 reviews (32 trials, 15 497 patients) had trustworthy evidence of clinically relevant effect on pain. Our grade assessment was moderate-to-high certainty of evidence. Analyses involved 528–6924 patients, almost all involving >1000 patients (Table 1).

**Table 1.** Pain intensity reduction and Patient Global Impression of Change for Groups 4 and 5

Drug, daily dose	Duration (weeks)	Number of participants in analyses	Percent with outcome		Number needed to treat (95%CI)	Risk difference (95% CI)
			Active	Placebo		
At least 50% pain intensity reduction						
Mirtazapine 15–45 mg	7–13	591	22	16	Not significantly different	0.05 (−0.01–0.12)
Duloxetine 60 mg	≤12	528	36	23	7.6 (4.8–18)	0.13 (0.06–0.21)
Duloxetine 120 mg	≤12	1234	36	21	6.9 (5.1–11)	0.14 (0.10–0.19)
Milnacipran 100 mg	≥8	1250	27	18	10 (7.0–20)	0.10 (0.05–0.15)
Milnacipran 200 mg	≥8	no data				
All SNRI, all doses	>6	6981	31	21	11(9–14)	0.09 (0.07–0.11)
Pregabalin 300 mg	8–14	1375	22	14	14 (8.9–32)	0.09 (0.04–0.14)
Pregabalin 450 mg	8–14	1874	24	14	9.7 (7.2–15)	0.09 (0.05–0.13)
Pregabalin 600 mg	8–14	1122	24	15	11 (7.1–21)	0.13 (0.07–0.18)
At least 30% pain intensity reduction						
Mirtazapine 15–45 mg	7–13	591	47	34	8 (5–20)	0.13 (0.05–0.21)
Duloxetine 60 mg	≤12	528	53	35	5.6 (3.8–10)	0.18 (0.10–0.26)
Duloxetine 120 mg	≤12	1030	42	30	8.5 (5.7–17)	0.16 (0.10–0.22)
Milnacipran 100 mg	≥8	1925	41	30	9 (6.5–15)	0.11 (0.07–0.16)
Milnacipran 200 mg	≥8	1798	39	29	10 (7.0–18)	0.10 (0.06–0.15)
SNRIs, all doses	>6	6924	40	32	10 (8–12)	0.10 (0.08–0.12)
Pregabalin 300 mg	8–14	1375	39	28	9.2 (6.3–17)	0.11 (0.06–0.16
Pregabalin 450 mg	8–14	1874	43	29	7.2 (5.5–10)	0.14 (0.10–0.18)
Pregabalin 600 mg	8–14	1122	39	28	9.4 (6.2–19)	0.11 (0.05–0.16)
Patient global impression of change: very much improved						
Mirtazapine 15–45 mg	7–13	Not calculated as only 40 patients				
Duloxetine 60 mg	≤12	only mean data				
Duloxetine 120 mg	≤12	only mean data				
Milnacipran 100 mg	≥8	only mean data				
Milnacipran 200 mg	≥8	only mean data				
SNRIs, all doses	>6	not reported				
Pregabalin 300 mg	8–14	1375	17	10	16 (10–37)	0.06 (0.03–0.10)
Pregabalin 450 mg	8–14	1869	17	9	12 (9–20)	0.08 (0.05–0.11)
Pregabalin 600 mg	8–14	1122	12	7	22 (13–89)	0.05 (0.01–0.08)
Patient global impression of change: much or very much improved						
Mirtazapine 15–45 mg	7–13	Not calculated as only 40 patients				
Duloxetine 60 mg	≤12	only mean data				
Duloxetine 120 mg	≤12	only mean data				
Milnacipran 100 mg	≥8	1925	38	25	7.8 (5.9–12)	0.13 (0.09–0.17)
Milnacipran 200 mg	≥8	1673	36	23	7.7 (5.8–12)	0.13 (0.09–0.17)
SNRIs, all doses	>6	2918	52	29	5.0 (4–8)	0.19 (0.12–0.26)
Pregabalin 300 mg	8–14	1375	36	27	11.0 (7.3–25)	0.09 (0.04–0.14)
Pregabalin 450 mg	8–14	1869 (5)	36	27	11.0 (7.8–22)	0.09 (0.05–0.13)
Pregabalin 600 mg	8–14	1122	40	27	7.8 (5.5–14)	0.13 (0.07–0.18)

NNT values not shown where results are not statistically significant.

SNRI: serotonin-norepinephrine reuptake inhibitor.

**Figure 2.**
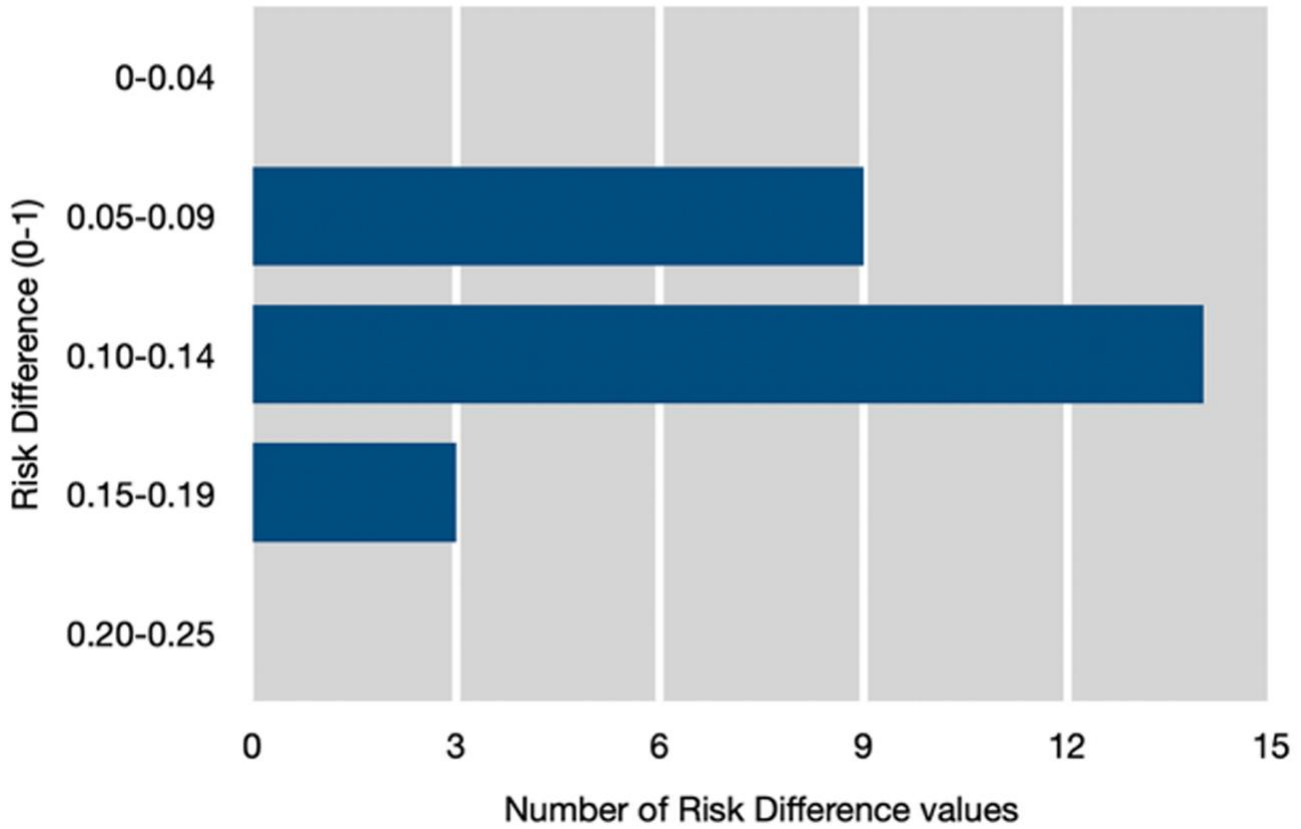
Risk differences found for all drugs and doses where there was trustworthy evidence (Groups 4 and 5) and for all pain outcomes

There was consistent evidence of benefit for pain intensity reduction and Patient Global Impression of Change (PGIC) for duloxetine (60 mg and 120 mg daily), milnacipran (100 mg and 200 mg daily), for all SNRIs combined, and for pregabalin (300 mg, 450 mg and 600 mg daily). There was considerable consistency in the effect size found, irrespective of the drug, dose or pain or improvement outcome used (Fig. 2, Table 1). Of the 26 results, 20 lay between RD 0.08 and RD 0.14, and six were only narrowly outside this range. The review of pregabalin also included two enriched enrolment randomized withdrawal (EERW) trials lasting 13 or 26 weeks, with an outcome of maintenance of therapeutic relief (defined as pain relief of at least 30%) which found the RR for pregabalin compared with placebo was 1.9 (95% CI 1.5–2.4) and NNT was 5.3 (95% CI 3.9–8.2).

PGIC data were less commonly reported (Table 1). Where PGIC was reported for ‘much or very much improved’ efficacy, estimates were broadly in line with those for pain reduction of ≥30%, and for ‘very much improved’ were broadly in line with pain reduction of ≥50%. PGIC results were unavailable for mirtazapine, duloxetine and SNRI.

#### Withdrawals and adverse events

Table 2 shows withdrawals and adverse events in the five reviews with largest amounts of data. Average adverse event withdrawals were between 6% and 9% for duloxetine, milnacipran and pregabalin. Serious adverse events were both rare and on no occasion were they statistically different between the active drug and placebo. Somnolence, dizziness and weight gain seemed common with pregabalin, and nausea with SNRIs.

**Table 2.** Risk difference for withdrawals and adverse events for Groups 4 and 5

	Mirtazapine 15–45 mg	Duloxetine 60 mg	Milnacipran 100 mg	SNRI (duloxetine and milnacipran)	Pregabalin 450 mg
Defined primary and secondary withdrawal and adverse event outcomes					
Tolerability: adverse event withdrawal	0.00 (−0.02–0.03)	0.06 (0.05–0.07)	0.07 (0.04–0.10)	0.07 (0.04–0.10)	0.09 (0.06–0.12)
	GRADE: Low				
Safety: serious adverse events	0.00 (−0.01–0.02)	0.00 (−0.01–0.0)	0.00 (−0.01–0.01)	0.00 (−0.01–0.0)	0.01 (−0.0–0.02)
	GRADE: Low				
Lack of efficacy withdrawal	0.01 (−0.01–0.02)		−0.03 (−0.05 to −0.0)	−0.01 (−0.04 to −0.01)	−0.07 (−0.09 to −0.04)
	GRADE: Low				
Other reported withdrawal and adverse event outcomes					
All cause withdrawal			0.04 (0.0–0.08)		0.02 (−0.02–0.06)
Reporting ≥1 adverse event	0.12 (−0.01–0.26)	0.10 (0.07–0.12)			0.17 (0.13–0.20)
Somnolence	0.24 (0.18–0.30)	0.08 (0.06–0.10)		0.05 (0.02–0.08)	0.19 (0.16–0.22)
Dizziness		0.04 (0.02–0.07)			0.30 (0.26–0.32)
Weight gain	0.17 (0.11–0.23)				0.09 (0.07–0.12)
Peripheral oedema					0.05 (0.03–0.07)
Nausea		0.14 (0.11–0.17)	0.16 (0.12–0.19)	0.16 (0.14–0.19)	
Insomnia				0.03 (0.01–0.04)	
Constipation			0.12 (0.09–0.14)		
Hot flush			0.08 (0.06–0.10)		
Dizziness			0.05 (0.03–0.08)		
Palpitations			0.05 (0.03–0.07)		
Increased heart rate/tachycardia		0.04 (0.03–0.06)			
Hyperhidrosis			0.06 (0.04–0.08)		
Vomiting			0.04 (0.01–0.06)		
Hypertension			0.05 (0.03–0.06)		
Elevated alanine aminotransferase (ALT)	0.13 (0.04–0.22)				
Dry mouth		0.07 (0.05–0.10)			

Note that adverse event results for duloxetine are those reported for a combined analysis of neuropathic pain and FMS, and those for SNRI are for all drugs and doses combined. Empty cells indicate no available data.

SNRI: serotonin and noradrenaline reuptake inhibitors.

#### Efficacy outcomes other than pain

Four reviews reported outcomes other than pain [14, 59, 61, 62]. None of the medications had a substantial effect size (SMD >0.2) except for SSRIs for depression and mirtazapine for sleep problems (Table 3).

**Table 3.** Sleep problems, fatigue, depressed mood and health-related quality of life for Groups 4 and 5

Medication	Review	Sleep problems SMD (95% CI)	Fatigue SMD (95% CI)	Depressed mood SMD (95% CI)	Health related quality of life SMD (95% CI)
SNRI (duloxetine, milnacipran)	[53]	−0.7 (−0.15–0.01)	−0.13 (−0.18 to −0.08)	−0.16 (−0.21 to −0.11)	−0.20 (−0.25 to −0.15)
SSRI (citalopram, fluoxetine, paroxetine)	[51]	0.03 (−0.26–0.31)	−0.26 (−0.55–0.03)	−0.39 (−0.65 to −0.14)	Only two studies with 70 participants
Mirtazapine	[52]	−0.23 (−0.39 to −0.06)	−0.02 (−0.19–0.16)	−0.67 (−1.44–0.10)	RD for 20% or more improvement: 0.08 (−0.01–0.16)

CI: confidence interval; RD: risk difference; SMD: standardized mean difference; SNRI: serotonin and noradrenaline reuptake inhibitors; SSRI: selective serotonin reuptake inhibitors.

There was a small improvement for participants receiving mirtazapine 15 mg to 45 mg a day in participant-reported sleep problems, but not in fatigue, negative mood, nor in health-related quality of life [52].

For duloxetine 60 mg and 120 mg daily [14], a significant benefit was reported at 12 weeks or less for the mean improvement in SF-36 physical subscore, bodily pain subscore, the Patient Reported Global Impression of Improvement, the British Pain Inventory severity of average pain, and pain at rest (night pain). Significant benefit for the mean improvement in SF-36 mental subscore was found only for the 120 mg dose.

For SNRIs (duloxetine and milnacipran) there were improvements overall and with each drug separately, for fatigue, depression, anxiety and HRQoL [51]. There was an overall improvement in the tender point threshold with duloxetine but not milnacipran. There was no overall improvement in patient-reported sleep problems, although there was an improvement with duloxetine when analysed independently.

## Discussion

Of 21 Cochrane reviews of pharmacological treatments for FMS, seven found no trials, seven found some trials but with insufficient data to form any conclusion and two had potential publication bias so could not be trusted. Only five reviews had trustworthy evidence of some or no effect. Effect sizes for the primary outcome of at least 50% pain intensity reduction were modest, with RD values of 0.09–0.14, and NNTs of 6.9–14 compared with placebo. Duloxetine, pregabalin and milnacipran were all capable of producing substantial pain relief lasting for at least 12 weeks in about 1 in 10 people with moderate or severe pain associated with FMS. Adverse events were common but serious adverse events were rare. EERW trials of pregabalin indicated that initial benefit (4–6 weeks) is likely to be maintained for six months, with no evidence that early failure was followed by later success. By contrast, more participants switched to ongoing placebo lost a therapeutic response compared with those receiving ongoing pregabalin [55].

The FDA licensed duloxetine (2008), milnacipran (2009) and pregabalin (2007) for the treatment of FMS in the United States. The European Union has not licensed these medications. A 2018 written answer to a question in the European Parliament explained that: ‘After careful examination, EMA [European Medicines Agency] was of the opinion that the benefits of these medicines in the treatment of fibromyalgia did not outweigh the risks and therefore recommended that the marketing authorisation be refused in this indication’ [66]. This applies also in the United Kingdom.

The Cochrane reviews were of good quality, with AMSTAR-2 ratings of high or moderate, contrasting with non-Cochrane systematic reviews; assessment of many systematic reviews of pain (including musculoskeletal pain) found >80% of them were of low or critically low quality [67]. Seventeen of the 21 reviews reported five or more of the eight required critical pain criteria, comparing well with an overview of systematic reviews for cannabinoid interventions for pain that found these criteria to be almost universally ignored [37].

A potential weakness was that some overview authors were also authors of some reviews, but data extraction and assessment were performed by uninvolved overview authors, and all authors were able to comment at all stages. The overview is in broad agreement with a previous Cochrane overview examining antiepileptic drugs for neuropathic pain and fibromyalgia [19]; other overviews found no other useful data [68, 69].

A strength of this overview is that participant demographics reflected those of people with FMS, predominantly women in their fifth and sixth decades of life, but with a preponderance of white people. A recognized set of diagnostic criteria was commonly used, though trials often excluded people with depression and/or anxiety or with more serious mental disorders. All studies excluded people with inflammatory rheumatic diseases. FMS is common in people with these diseases, so this represents a major weakness of the pharmacological studies, as their populations were not representative of patients in routine clinical care.

Most of the reviews examined pain as a primary outcome. Pain is a presenting symptom, but it is well recognized that there are many other problematic concomitant symptoms, including fatigue, depression and sleep disturbance. The symptom burden often precludes work and leads to a substantial reduction in quality of life. Only average changes in these symptoms were reported, and averages showed no substantial benefit for these other key symptoms of FMS, where measures were assessed. Analysis at the individual patient level could provide a more relevant analysis. Individual patient level analysis of pregabalin trial data shows that improvement in pain is linked to improvement in symptoms such as quality of life and work [22–24]. Outcome measures for different pain measures and other symptoms show similar effect sizes [70]. This type of analysis is missing for duloxetine and milnacipran.

We know of no large body of evidence that would usefully have added to the completeness of the overview, except for a report of three large trials (3864 participants) comparing mirogabalin, placebo and pregabalin 300 mg [71]. It showed that mirogabalin had comparable efficacy to pregabalin in those trials (RD 0.09 for mirogabalin 15 mg daily and 0.14 for pregabalin 300 mg daily for PGIC score of 2 or below; Supplementary Data S11, available at *Rheumatology* online).

Duloxetine, milnacipran and pregabalin have good evidence of efficacy in a small proportion of patients and might be recommended for use in primary care for a trial period of 4–6 weeks. Treatment should cease if substantial pain relief is not experienced by six weeks. Because relatively few trial participants achieved substantial pain relief with duloxetine, milnacipran and pregabalin, it is important to establish stopping rules, so that when someone does not respond within a specified time, they can be switched to a suitable alternative treatment. This would reduce the number of individuals exposed to adverse events from medications in the absence of benefit. Evidence from this overview does not support guidelines recommending amitriptyline at doses below 50 mg daily [69, 72], cyclobenzaprine and tramadol [9].

The combination of this overview of pharmacological interventions, together with a previous overview examining non-pharmacological interventions [3], had data from 31 Cochrane reviews, representing 268 clinical trials and the involvement of over 29 000 patients with FMS. It is disappointing that 27 of those reviews had no, or inadequate, information that might help guide therapy.

## Conclusions

There is moderate-to-good evidence that duloxetine, milnacipran and pregabalin provide substantial pain relief for a small proportion (around 1 in 10) of adults with moderate or severe FMS pain for 4–12 weeks. There is no evidence about which adults with FMS might benefit from the medications or might experience adverse events. There was no trustworthy evidence for carbamazepine, clonazepam, lamotrigine, phenytoin, oxycodone, topiramate, valproate, antipsychotics, cannabinoids, combination therapy, gabapentin, lacosamide, MAOIs, NSAIDs, amitriptyline or SSRIs.

## Supplementary Material

keae707_Supplementary_Data

## Data Availability

The data underlying this article are available in the article and in its online supplementary material.
