# Supplementary material for: Effectiveness of pharmacological therapies for fibromyalgia syndrome in adults: an overview of Cochrane Reviews
Source: Rheumatology (Oxford). 2024 Dec 20;64(5):2385–94. doi: 10.1093/rheumatology/keae707 (PMC12048062; doi:10.1093/rheumatology/keae707)
Supplement: keae707_Supplementary_Data [file keae707_supplementary_data.pdf]

Effectiveness of pharmacological therapies for fibromyalgia syndrome in adults: an overview of Cochrane Reviews: Supplementary files

Table of contents

Supplementary data S1: Protocol amendments and methods details

Supplementary data S2: PRIOR statement

Supplementary data S3: Outcomes assessed in the reviews

Supplementary data S4: Quality assessment of eligible reviews

Supplementary data S5: List of excluded reviews with reason

Supplementary table S6: Details of included reviews - drug names, review status, diagnostic criteria, age, sex and initial pain measures

Supplementary table S7: Included reviews - details of the date of search, review group, main pain and other outcomes, number of trials and participants included, and number in main pain analysis

Supplementary table S8: AMSTAR-2 scores for the included studies

Supplementary table S9: Scoring the critical pain criteria

Supplementary table S10: Grade assessment by review and overview authors compared

Supplementary data S11: Calculations for mirogabalin efficacy for pain relief

Supplementary data S1: Protocol amendments and methods details

Minor amendments were made to the protocol because of changes to team membership and to include the pain critical elements that we had introduced. There were also minor textual changes, but no changes to the methods. The following text was included in the protocol.

13 August 2021<br>New citation: no major change	Addition of AMSTAR-2 and pain critical items to evaluations of reviews to be included in this overview.<br>New author team to align with parallel overview review on non-pharmacological interventions.<br>Minor changes in text to bring the protocol in line with current standards and to update with more recent relevant publications.<br>Title updated to include 'fibromyalgia syndrome'.

Eligibility outcomes.

Serious adverse events typically include any untoward medical occurrence or effect that at any dose results in death, is life-threatening, requires hospitalisation or prolongation of existing hospitalisation, results in persistent or significant disability or incapacity, is a congenital anomaly or birth defect, is an 'important medical event' that may jeopardise the person, or may require an intervention to prevent one of the above characteristics or consequences (1).

Search strategy

The following strategy was run in the Cochrane Database of Systematic Reviews in the Cochrane Library (2021 issue 12) on 1 January 2022.

#1 MeSH descriptor: [Fibromyalgia] explode all trees and with qualifier(s): [Drug therapy - DT]
#2 "fibromyalgia":ti (Word variations have been searched)
#3 #1 or #2

No date or language limits were applied to the strategy.

The Cochrane Library was searched again 1 May 2024.

Data extraction and data management

We extracted information from randomised studies for the following measures or calculated them from available data:

- risk difference (RD), or risk ratio (RR)
- number needed to treat for an additional beneficial outcome (NNTB)
- number needed to treat to prevent an event (NNTp)
- number needed to treat for an additional harmful outcome (NNTH).

We anticipated that reviews might have used a variety of outcome measures, with the majority using standard subjective scales (numerical rating scales or visual analogue scales) for pain intensity or pain relief, or both. We were particularly interested in the Initiative on Methods, Measurement, and Pain Assessment in Clinical Trials (IMMPACT) definitions for moderate and substantial benefit in chronic pain studies (2). These are defined as at least 30% pain relief over baseline (moderate), at least 50% pain relief over baseline (substantial), much or very much improved on Patient Global Impression of Change (PGIC), (moderate), and very much improved on PGIC (substantial). These dichotomous outcomes are used as

pain responses often do not follow a normal (Gaussian) distribution. People with chronic pain desire high levels of pain relief, ideally more than 50%, and with residual pain not worse than mild (3, 4).

Rationale for the choice of AMSTAR 2

A 1996 survey indicated that 90% of meta-analyses of analgesic interventions had methodological flaws that could limit their validity, and that meta-analyses of low quality produced significantly more positive conclusions (5). Even in good quality Cochrane reviews, the use of Grading of Recommendations Assessment, Development and Evaluation (GRADE) to summarise the certainty of evidence indicates that fewer than 20% of reviews actually have any high-quality evidence (6). Judging systematic review quality is difficult. AMSTAR (A MeaSurement Tool to Assess systematic Reviews) is most often used, and a newer version now exists in AMSTAR-2 (7). This is a generic tool examining what is regarded as best practice in systematic review methodology, and its use indicated that 86% of systematic reviews provided low or critically low confidence in their results in cannabis-based medicine reviews for pain (8), 90% in exercise therapy for low back pain (9), 99% in bariatrics (10), and 100% in spine surgery (11). Cochrane reviews rated mainly moderate or high confidence (8, 9).

GRADE criteria for assigning grade of evidence

The GRADE system uses the following criteria to assign a quality level to a body of evidence (12):

1. High: randomised trials; or double-upgraded observational studies
2. Moderate: downgraded randomised trials; or upgraded observational studies
3. Low: double-downgraded randomised trials; or observational studies
4. Very low: triple-downgraded randomised trials; or downgraded observational studies; or case series/case reports

Factors that may decrease the quality level of a body of evidence are:

1. limitations in the design and implementation of available studies suggesting high likelihood of bias;
2. indirectness of evidence (indirect population, intervention, control, outcomes);
3. unexplained heterogeneity or inconsistency of results (including problems with subgroup analyses);
4. imprecision of results (wide confidence intervals);
5. high probability of publication bias.

Factors that may increase the quality level of a body of evidence are:

1. large magnitude of effect;
2. all plausible confounding would reduce a demonstrated effect or suggest a spurious effect when results show no effect;
3. dose-response gradient.

We paid particular attention to inconsistency, where point estimates vary widely across studies, or confidence intervals of studies showing minimal or no overlap (13). Small studies have been shown to overestimate treatment effects, probably because the conduct of small studies is more likely to be less rigorous, allowing critical criteria to be compromised (14, 15), and large studies often have smaller treatment effects (16).

In addition, there may be circumstances where the overall rating for a particular outcome needs to be adjusted as recommended by GRADE guidelines (12, 17). For example, if there are so few data that the results are highly susceptible to the random play of chance, or if studies use last observation carried forward (LOCF) imputation in circumstances where there are substantial differences in adverse event withdrawals, one would have no confidence in the result, and would need to downgrade the certainty of the evidence by three levels, to very low certainty. In circumstances where there were no data reported for an outcome, we reported the level of evidence as very low certainty (17).

References

1. European Medicines Agency. NOTE FOR GUIDANCE ON CLINICAL SAFETY DATA MANAGEMENT: DEFINITIONS AND STANDARDS FOR EXPEDITED REPORTING. European Medicines Agency: London, 1995.
2. Dworkin RH, Turk DC, Wyrwich KW, Beaton D, Cleeland CS, Farrar JT, et al. Interpreting the clinical importance of treatment outcomes in chronic pain clinical trials: IMMPACT recommendations. *J Pain* 2008;9:105-21.
3. Moore RA, Straube S, Aldington D. Pain measures and cut-offs - 'no worse than mild pain' as a simple, universal outcome. *Anaesthesia* 2013;68:400-12.
4. O'Brien EM, Staud RM, Hassinger AD, McCulloch RC, Craggs JG, Atchison JW, . Patient-centered perspective on treatment outcomes in chronic pain. *Pain Med* 2010;11:6-15.
5. Jadad AR, McQuay HJ. Meta-analyses to evaluate analgesic interventions: a systematic qualitative review of their methodology. *J Clin Epidemiol* 1996;49:235-43.
6. Fleming PS, Koletsi D, Ioannidis JP, Pandis N. High quality of the evidence for medical and other health-related interventions was uncommon in Cochrane systematic reviews. *J Clin Epidemiol* 2016;78:34-42.
7. Shea BJ, Reeves BC, Wells GA, Thuku M, Hamel C, Moran J, Moher D, et al. AMSTAR 2: a critical appraisal tool for systematic reviews that include randomised or non-randomised studies of healthcare interventions, or both. *BMJ* 2017;358:j4008.
8. Moore RA, Fisher E, Finn DP, et al. Cannabinoids, cannabis, and cannabis-based medicines for pain management: an overview of systematic reviews. *Pain* 2021; 162(Suppl 1): S67-S79. DOI: 10.1097/j.pain.0000000000001941
9. Almeida MO, Yamato TP, Parreira PDCS, Costa LOP, Kamper S, Saragiotto BT. Overall confidence in the results of systematic reviews on exercise therapy for chronic low back pain: a cross-sectional analysis using the Assessing the Methodological Quality of Systematic Reviews (AMSTAR) 2 tool. *Braz J Phys Ther* 2020; 24:103-17.
10. Storman M, Storman D, Jasinska KW, Swierz MJ, Bala MM. The quality of systematic reviews/meta-analyses published in the field of bariatrics: A cross-sectional systematic survey using AMSTAR 2 and ROBIS. *Obes Rev* 2020;21: e12994.
11. Dettori JR, Skelly AC, Brodt ED. Critically low confidence in the results produced by spine surgery systematic reviews: an AMSTAR-2 evaluation from 4 spine journals. *Glob Spine J* 2020;10:667-73.
12. Schünemann HJ, Higgins JPT, Vist GE, et al. Chapter 14: Completing 'Summary of findings' tables and grading the certainty of the evidence. In: Higgins Jpt TJCJCMLTPMJWVA, (ed.) *Cochrane Handbook for Systematic Reviews of Interventions version 6.2 (updated February 2021)*. Cochrane, 2021.
13. Guyatt GH, Oxman AD, Kunz R, Woodcock J, Brozek J, Helfand M, et al. GRADE guidelines: 7. Rating the quality of evidence--inconsistency. *J Clin Epidemiol* 2011;64:1294-302.

14. Dechartres A, Trinquart L, Boutron I, Ravaud P. Influence of trial sample size on treatment effect estimates: meta-epidemiological study. *BMJ* 2013;346:f2304.
15. Nuesch E, Trelle S, Reichenbach S, Rutjes AW, Tschannen B, Altman DG, et al. Small study effects in meta-analyses of osteoarthritis trials: meta-epidemiological study. *BMJ* 2010;341:c3515
16. Dechartres A, Altman DG, Trinquart L, Boutron I, Ravaud P. Association between analytic strategy and estimates of treatment outcomes in meta-analyses. *JAMA* 2014;312:623-30.
17. Guyatt G, Oxman AD, Sultan S, Brozek J, Glasziou P, Alonso-Coello P, et al. GRADE guidelines: 11. Making an overall rating of confidence in effect estimates for a single outcome and for all outcomes. *J Clin Epidemiol* 2013;66:151-7.

Supplementary data S2: PRIOR statement

Section topic	Item No	Item	Location where item is reported
**Title**			
Title	1	Identify the report as an overview of reviews.	Title
**Abstract**			
Abstract	2	Provide a comprehensive and accurate summary of the purpose, methods, and results of the overview of reviews.	Abstract
**Introduction**			
Rationale	3	Describe the rationale for conducting the overview of reviews in the context of existing knowledge.	Background
Objectives	4	Provide an explicit statement of the objective(s) or question(s) addressed by the overview of reviews.	Objectives
**Methods**			
Eligibility criteria	5a	Specify the inclusion and exclusion criteria for the overview of reviews. If supplemental primary studies were included, this should be stated, with a rationale.	Methods
	5b	Specify the definition of "systematic review" as used in the inclusion criteria for the overview of reviews.	Methods
Information sources	6	Specify all databases, registers, websites, organisations, reference lists, and other sources searched or consulted to identify systematic reviews and supplemental primary studies (if included). Specify the date when each source was last searched or consulted.	Search section in S1
Search strategy	7	Present the full search strategies for all databases, registers and websites, such that they could be reproduced. Describe any search filters and limits applied.	S1
Selection process	8a	Describe the methods used to decide whether a systematic review or supplemental primary study (if included) met the inclusion criteria of the overview of reviews.	Review processing section<br><br>Protocol
	8b	Describe how overlap in the populations, interventions, comparators, and/or outcomes of systematic reviews was identified and managed during study selection.	Methods
Data collection process	9a	Describe the methods used to collect data from reports.	Methods
	9b	If applicable, describe the methods used to identify and manage primary study overlap at the level of the comparison and outcome during data collection. For each outcome, specify the method used to illustrate and/or quantify the degree of primary study overlap across systematic reviews.	Not considered

	9c	If applicable, specify the methods used to manage discrepant data across systematic reviews during data collection.	Not considered
Data items	10	List and define all variables and outcomes for which data were sought. Describe any assumptions made and/or measures taken to identify and clarify missing or unclear information.	S3<br><br>Protocol
Risk of bias assessment	11a	Describe the methods used to assess risk of bias or methodological quality of the included systematic reviews.	Methods<br><br>S4
	11b	Describe the methods used to collect data on (from the systematic reviews) and/or assess the risk of bias of the primary studies included in the systematic reviews. Provide a justification for instances where flawed, incomplete, or missing assessments are identified but not reassessed.	Cochrane reviews used Cochrane RoB methods
	11c	Describe the methods used to assess the risk of bias of supplemental primary studies (if included).	Not applicable
Synthesis methods	12a	Describe the methods used to summarise or synthesise results and provide a rationale for the choice(s).	Data synthesis
	12b	Describe any methods used to explore possible causes of heterogeneity among results.	Not applicable in an overview like this looking at different drugs and doses.
	12c	Describe any sensitivity analyses conducted to assess the robustness of the synthesised results.	Not necessary since no pooling
Reporting bias assessment	13	Describe the methods used to collect data on (from the systematic reviews) and/or assess the risk of bias due to missing results in a summary or synthesis (arising from reporting biases at the levels of the systematic reviews, primary studies, and supplemental primary studies, if included).	Publication bias in Methods
Certainty assessment	14	Describe the methods used to collect data on (from the systematic reviews) and/or assess certainty (or confidence) in the body of evidence for an outcome.	Summary of findings – GRADE<br><br>S8 and S9
		**Results**	
Systematic review and supplemental primary study selection	15a	Describe the results of the search and selection process, including the number of records screened, assessed for eligibility, and included in the overview of reviews, ideally with a flow diagram.	Figure 1<br>Results, S5
	15b	Provide a list of studies that might appear to meet the inclusion criteria, but were excluded, with the main reason for exclusion.	S5
Characteristics of systematic reviews and supplemental primary studies	16	Cite each included systematic review and supplemental primary study (if included) and present its characteristics.	Description of included reviews<br><br>S6, S7

Primary study overlap	17	Describe the extent of primary study overlap across the included systematic reviews.	Results
Risk of bias in systematic reviews, primary studies, and supplemental primary studies	18a	Present assessments of risk of bias or methodological quality for each included systematic review.	Results<br><br>S8, S9
	18b	Present assessments (collected from systematic reviews or assessed anew) of the risk of bias of the primary studies included in the systematic reviews.	Results
	18c	Present assessments of the risk of bias of supplemental primary studies (if included).	Not applicable
Summary or synthesis of results	19a	For all outcomes, summarise the evidence from the systematic reviews and supplemental primary studies (if included). If meta-analyses were done, present for each the summary estimate and its precision and measures of statistical heterogeneity. If comparing groups, describe the direction of the effect.	Tables 1-3
	19b	If meta-analyses were done, present results of all investigations of possible causes of heterogeneity.	Not applicable
	19c	If meta-analyses were done, present results of all sensitivity analyses conducted to assess the robustness of synthesised results.	Not applicable
Reporting biases	20	Present assessments (collected from systematic reviews and/or assessed anew) of the risk of bias due to missing primary studies, analyses, or results in a summary or synthesis (arising from reporting biases at the levels of the systematic reviews, primary studies, and supplemental primary studies, if included) for each summary or synthesis assessed.	We did not assess reporting biases because it is not helpful (see, for example: Thornton A, Lee P. Publication bias in meta-analysis: its causes and consequences. J Clin Epidemiol. 2000 Feb;53(2):207-16. doi: 10.1016/s0895-4356(99)00161-4.)
Certainty of evidence	21	Present assessments (collected or assessed anew) of certainty (or confidence) in the body of evidence for each outcome.	Effects of interventions: pain<br><br>Table 1
**Discussion**			
Discussion	22a	Summarise the main findings, including any discrepancies in findings across the included systematic reviews and supplemental primary studies (if included).	Discussion
	22b	Provide a general interpretation of the results in the context of other evidence.	Overall completeness and applicability of

			evidence discussed
	22c	Discuss any limitations of the evidence from systematic reviews, their primary studies, and supplemental primary studies (if included) included in the overview of reviews. Discuss any limitations of the overview of reviews methods used.	Quality of the evidence discussed<br><br>Potential biases in the overview process discussed
	22d	Discuss implications for practice, policy, and future research (both systematic reviews and primary research). Consider the relevance of the findings to the end users of the overview of reviews, eg, healthcare providers, policymakers, patients, among others.	Discussion
**Other information**			
Registration and protocol	23a	Provide registration information for the overview of reviews, including register name and registration number, or state that the overview of reviews was not registered.	Methods
	23b	Indicate where the overview of reviews protocol can be accessed, or state that a protocol was not prepared.	Methods
	23c	Describe and explain any amendments to information provided at registration or in the protocol. Indicate the stage of the overview of reviews at which amendments were made.	S1
Support	24	Describe sources of financial or non-financial support for the overview of reviews, and the role of the funders or sponsors in the overview of reviews.	Funding sources
Competing interests	25	Declare any competing interests of the overview of reviews' authors.	Declaration of interests
Author information	26a	Provide contact information for the corresponding author.	Corresponding author
	26b	Describe the contributions of individual authors and identify the guarantor of the overview of reviews.	Contributions of authors
Availability of data and other materials	27	Report which of the following are available, where they can be found, and under which conditions they may be accessed: template data collection forms; data collected from included systematic reviews and supplemental primary studies; analytic code; any other materials used in the overview of reviews.	Manuscript Appendices

Supplementary data S3: Outcomes assessed in the overview

Primary outcomes	Details/examples
Participant-reported pain relief of 50% or greater	This was characterised as a substantial improvement
Patient Global Impression of Change (PGIC)	A report of ‘very much improved’ was characterised as a substantial improvement
Number of participants experiencing any serious adverse event.	
Withdrawals due to adverse events	This is measuring tolerability
**Secondary outcomes**	
Participant-reported pain relief of 30% or greater (moderate improvement).	This was characterised as moderate improvement
PGIC report of ‘much improved’	
Participant-reported sleep problems.	Measured as a continuous outcome: we preferred composite measures over single item scales
Participant-reported fatigue.	Measured as a continuous outcome: we preferred composite measures over single item scales
Participant-reported mean pain intensity.	Measured as a continuous outcome: we preferred change from baseline scores over intensity at the end of the study
Participant-reported health-related quality of life.	We preferred disease-specific instruments such as the Fibromyalgia Impact Questionnaire (FIQ) over generic instruments. If authors reported FIQ scores, we calculated the number of participants with a clinically relevant improvement of 20% or greater.
Participant-reported negative mood.	Measured using continuous outcome: we preferred composite measures such as the Beck Depression Inventory (BDI) or the Hospital Anxiety and Depression (HAD) scale over single item scales.
Withdrawals due to lack of efficacy.	
Participants with any adverse event.	
Participants with specific adverse events, for example somnolence, substantial weight gain, elevated liver enzymes	

Supplementary data S4: Quality assessment of eligible Cochrane reviews

**Quality assessment tools/additional questions**
AMSTAR-2 tool (34)
Were studies included in the review both randomised and double blind? (36)
Did the review user-defined diagnostic criteria for fibromyalgia?
Did the review include only studies in which patients made their own assessment of pain? (37)<br><br>Professional and patient assessment often disagrees, with professionals significantly underestimating pain.
Did the review use studies with defined minimum pain intensity of moderate or severe pain? (1)<br><br>Mild pain at baseline can reduce the sensitivity of trials to demonstrate an analgesic effect
Did the review examine study size as a confounding factor in any analysis of efficacy?<br><br>Systematic reviews have been criticised for being overconfident of results with inadequate data (38-40); there is increasing evidence of the importance of small trial size, both because of random chance, and as an important source of bias (41-44).
Did the review examine susceptibility to publication bias?
Did the review examine or comment upon imputation methods for missing data as a potential source of bias?
Did the review analyse the inclusion and exclusion criteria of the studies and discuss the utility of the study results to the people with fibromyalgia in routine clinical care?
Did the review analyse if the studies analysed the impact of the use of rescue medication on study findings?

Supplementary data S5: List of excluded reviews and reason for exclusion

Reference	Reason for exclusion
Bernardy K, Klose P, Busch AJ, et al. Cognitive behavioural therapies for fibromyalgia. *Cochrane Database Syst Rev* 2013; 2013(9): CD009796. DOI: 10.1002/14651858.CD009796.pub2	Not pharmacological
Bidonde J, Busch AJ, Schachter CL, et al. Aerobic exercise training for adults with fibromyalgia. *Cochrane Database Syst Rev* 2017; 6(6): CD012700. DOI: 10.1002/14651858.CD012700	Not pharmacological
Bidonde J, Busch AJ, Schachter CL, et al. Mixed exercise training for adults with fibromyalgia. *Cochrane Database Syst Rev* 2019; 5(5): CD013340. DOI: 10.1002/14651858.CD013340	Not pharmacological
Bidonde J, Busch AJ, van der Spuy I, et al. Whole body vibration exercise training for fibromyalgia. *Cochrane Database Syst Rev* 2017; 9(9): CD011755. DOI: 10.1002/14651858.CD011755.pub2	Not pharmacological
Bidonde J, Busch AJ, Webber SC, et al. Aquatic exercise training for fibromyalgia. *Cochrane Database Syst Rev* 2014; 2014(10): CD011336. DOI: 10.1002/14651858.CD011336	Not pharmacological
Busch AJ, Barber KA, Overend TJ, et al. Exercise for treating fibromyalgia syndrome. *Cochrane Database Syst Rev* 2007; (4): CD003786. DOI: 10.1002/14651858.CD003786.pub2	Not pharmacological
Busch AJ, Webber SC, Richards RS, et al. Resistance exercise training for fibromyalgia. *Cochrane Database Syst Rev* 2013; 2013(12): CD010884. DOI: 10.1002/14651858.CD010884	Not pharmacological
Cipriani A, Koesters M, Furukawa TA, et al. Duloxetine versus other anti-depressive agents for depression. *Cochrane Database Syst Rev* 2012; 10(10): CD006533. DOI: 10.1002/14651858.CD006533.pub2	Depression: out of scope
Cooper TE, Wiffen PJ, Heathcote LC, et al. Antiepileptic drugs for chronic non-cancer pain in children and adolescents. *Cochrane Database Syst Rev* 2017; 8(8): CD012536. DOI: 10.1002/14651858.CD012536.pub2	Children; out of scope
Deare JC, Zheng Z, Xue CC, et al. Acupuncture for treating fibromyalgia. *Cochrane Database Syst Rev* 2013; 2013(5): CD007070. DOI: 10.1002/14651858.CD007070.pub2	Not pharmacological
Derry S, Phillips T, Moore RA, et al. Milnacipran for neuropathic pain in adults. *Cochrane Database Syst Rev* 2015; 2015(7): CD011789. DOI: 10.1002/14651858.CD011789	Not FMS
Fisher E, Law E, Dudeney J, et al. Psychological therapies for the management of chronic and recurrent pain in children and adolescents. *Cochrane Database Syst Rev* 2018; 9(9): CD003968. DOI: 10.1002/14651858.CD003968.pub5	Children; out of scope
Galizia I, Oldani L, Macritchie K, et al. S-adenosyl methionine (SAMe) for depression in adults. *Cochrane Database Syst Rev* 2016; 10(10): CD011286. DOI: 10.1002/14651858.CD011286.pub2	Depression: out of scope
Gaskell H, Derry S, Stannard C, et al. Oxycodone for neuropathic pain in adults. *Cochrane Database Syst Rev* 2016; 7(7): CD010692. DOI: 10.1002/14651858.CD010692.pub3	Not FMS
Geneen LJ, Moore RA, Clarke C, et al. Physical activity and exercise for chronic pain in adults: an overview of Cochrane Reviews. *Cochrane Database of Systematic Reviews* 2017; (1).	Not pharmacological; overview
Johnson MI, Claydon LS, Herbison GP, et al. Transcutaneous electrical nerve stimulation (TENS) for fibromyalgia in adults. *Cochrane Database Syst Rev* 2017; 10(10): CD012172. DOI: 10.1002/14651858.CD012172.pub2	Not pharmacological
Karjalainen KA, Malmivaara A, van Tulder MW, et al. Multidisciplinary rehabilitation for fibromyalgia and musculoskeletal pain in working age adults. *Cochrane Database of Systematic Reviews* 1999; (3).	Not pharmacological
Khaliq W, Alam S, Puri NK. WITHDRAWN: Topical lidocaine for the treatment of postherpetic neuralgia. *Cochrane Database Syst Rev* 2013; 2013(10): CD004846. DOI: 10.1002/14651858.CD004846.pub3	Withdrawn; out of scope
Kim SY, Busch AJ, Overend TJ, et al. Flexibility exercise training for adults with fibromyalgia. *Cochrane Database Syst Rev* 2019; 9(9): CD013419. DOI: 10.1002/14651858.CD013419	Not pharmacological
Moore RA, Derry S, Aldington D, et al. Amitriptyline for neuropathic pain in adults. *Cochrane Database Syst Rev* 2015; 2015(7): CD008242. DOI: 10.1002/14651858.CD008242.pub3	Not FMS
Nnoaham KE, Kumbang J. WITHDRAWN: Transcutaneous electrical nerve stimulation (TENS) for chronic pain. *Cochrane Database Syst Rev* 2014; (7): CD003222. DOI: 10.1002/14651858.CD003222.pub3	Not pharmacological
O'Connell NE, Marston L, Spencer S, et al. Non-invasive brain stimulation techniques for chronic pain. *Cochrane Database of Systematic Reviews* 2018; (4).	Not pharmacological
Seidel S, Aigner M, Ossege M, et al. Antipsychotics for acute and chronic pain in adults. *Cochrane Database Syst Rev* 2013; 2013(8): CD004844. DOI: 10.1002/14651858.CD004844.pub3	Originally included, but no reference to FMS found
Theadom A, Cropley M, Smith HE, et al. Mind and body therapy for fibromyalgia. *Cochrane Database Syst Rev* 2015; 2015(4): CD001980. DOI: 10.1002/14651858.CD001980.pub3	Not pharmacological
Uceyler N, Sommer C, Walitt B, et al. WITHDRAWN: Anticonvulsants for fibromyalgia. *Cochrane Database Syst Rev* 2017; 10(10): CD010782. DOI: 10.1002/14651858.CD010782.pub2	Withdrawn
Wiffen PJ, Derry S, Bell RF, et al. Gabapentin for chronic neuropathic pain in adults. *Cochrane Database Syst Rev* 2017; 6(6): CD007938. DOI: 10.1002/14651858.CD007938.pub4	Not FMS
Wiffen PJ, Derry S, Moore RA, et al. Antiepileptic drugs for neuropathic pain and fibromyalgia - an overview of Cochrane reviews. *Cochrane Database Syst Rev* 2013; 2013(11): CD010567. DOI: 10.1002/14651858.CD010567.pub2	Overview, now superseded
Williams ACC, Fisher E, Hearn L, et al. Psychological therapies for the management of chronic pain (excluding headache) in adults. *Cochrane Database Syst Rev* 2020; 8(8): CD007407. DOI: 10.1002/14651858.CD007407.pub4	Not pharmacological

Supplementary table S6: Details of included reviews, showing drug names, review status, diagnostic criteria, mean age, sex and initial pain measures

Review	Drug	Review status	Diagnostic criteria	Mean age (years)	Sex (% Female)	Initial pain (0-10 scale)
**Group 1: Medicines and doses with no information**						
Birse 2012	Phenytoin	No update planned	Not defined	NS	NS	NS
Corrigan 2012	Clonazepam	No update planned	Not defined	NS	NS	NS
Gaskell 2016a	Oxycodone	No update planned	ACR 1990/2010	NS	NS	NS
Gill 2011	Valproate	No update planned	Not defined	NS	NS	NS
Wiffen 2013b	Lamotrigine	No update planned	Not defined	NS	NS	NS
Wiffen 2013c	Topiramate	No update planned	Not defined	NS	NS	NS
Wiffen 2014	Carbamazepine	No update planned	Not defined	NS	NS	NS
**Group 2: Medicines and doses with inadequate amounts of evidence**						
Cooper 2017a	Gabapentin	No update planned	ACR 1990/2010	48	90	≥4/10
Derry 2017	Nonsteroidal anti-inflammatory drugs	Up to date	ACR 1990/2010	39-50	89-100	6-7.5
Hearn 2012	Lacosamide	Update pending	Not defined	50	93	≥5/10
Thorpe 2018	Combinations of analgesics	Up to date	ACR 1990/2010	NR	NR	NR
Tort 2012	MAOI	No update planned	Any recognised criterion	39-49	85/100	≥4
Walitt 2016b	Cannabinoids	Up to date	ACR 1990/2010	50	81-93	NR
Walitt 2016a	Antipsychotics (Quetiapine)	Up to date	ACR 1990/2010	48-50	95-100	NR

Review	Drug	Review status	Diagnostic criteria	Mean age (years)	Sex (% Female)	Initial pain (0-10 scale)
**Group 3: Medicines and doses potentially subject to publication bias**						
Moore 2015b	Amitriptyline	No update planned	ACR 1990/2010	41-49	95	≥4/10
Walitt 2015	Selective serotonin reuptake inhibitors	Up to date	Any recognised	43-53	Most ≥95%	Mostly ≥4/10
**Group 4: Medicines and doses with trustworthy evidence of no effect on pain**						
Welsch 2018b	Mirtazapine	No update planned	ACR 1990/2010	44	86-100	≥4/10
**Group 5: Medicines and doses with trustworthy evidence of clinically relevant effect on pain**						
Cording 2015	Milnacipran	Up to date	ACR 1990/2010	47-50	92-97	≥4/10
Derry 2016	Pregabalin	No update planned	ACR 1990/2010	47-50	89-95	Baseline 6.5-7.8/10
Lunn 2014	Duloxetine	Update planned but inactive	ACR 1990/2010	NR	90-100	≥4/10
Welsch 2018a	Serotonin-norepinephrine reuptake inhibitors	Up to date	"any published, recognized and standardized criteria"	47-55	82-100	Generally, ≥3/10

ACR = American College of Rheumatology; NS = no studies; NR = not reported

Supplementary table S7: Included reviews, with details of the date of last search, review group, main pain and other outcomes, the total of trials and participants included, and the totals for the main pain analysis

						Number included		Number included in the main pain analysis	
Drug	Author, year	Date of last search	Review Group	Main primary Pain measures	Other measures	Trials	Participants	Trials	Participants
**Antidepressants**									
Amitriptyline	Moore 2015	Mar-15	PaPaS	PR/ PGIC	W and AE	9	649	4	275
Duloxetine	Lunn 2014	Apr-13	NMD	PR/ PGIC	W and AE	6	2249	4	1673
MAOI	Tort 2012	Nov-10	MSG	P	AE and 11 others	2	230	2	121
Milnacipran	Cording 2015	May-15	PaPaS	PR/ PGIC	W and AE	7	4000	3	1925
Mirtazapine	Welsch 2018b	Jul-17	PaPaS	PR/PGIC	AE, AEW, SAE, sleep, fatigue, depression, others	3	606	3	591
SNRIs: desvenlexafine, duloxetine, milnacipran	Welsch 2018a	Aug-17	PaPaS	PR/ PGIC	W and AE	18	7903	15	6918
SSRIs: citalopram, escitalopram, fluoxetine, fluvoxamine, paroxetine, sertraline	Walitt 2015	Jun-14	MSG	PR/PGIC	Fatigue, sleep, depression, W, AE	7	383	6	343
**Antiepileptics**									
Carbamazepine	Wiffen 2014	Feb-14	PaPaS	PR/ PGIC	W and AE	0	0	0	0
Clonazepam	Corrigan 2012	Feb-12	PaPaS	PR/ PGIC	W and AE	0	0	0	0
Gabapentin	Cooper 2017a	May-16	PaPaS	PR/ PGIC	W and AE	1	150	1	150
Lacosamide	Hearn 2012	Aug-11	PaPaS	PR/ PGIC	W and AE	1	159	1	159
Lamotrigine	Wiffen 2013b	Aug-13	PaPaS	PR/ PGIC	W and AE	0	0	0	0

						Number included		Number included in the main pain analysis	
Drug	Author, year	Date of last search	Review Group	Main primary Pain measures	Other measures	Trials	Participants	Trials	Participants
Phenytoin	Birse 2012	Feb-12	PaPaS	PR/ PGIC	W and AE	0	0	0	0
Pregabalin	Derry 2016	Mar-16	PaPaS	PR/ PGIC	W and AE	8	4152	5	1875
Topiramate	Wiffen 2013c	May-13	NMD	PR/ PGIC	W and AE	0	0	0	0
Valproate	Gill 2011	Jun-11	PaPaS	PR/ PGIC	W and AE	0	0	0	0
**Antipsychotics**									
Quetiapine	Walitt 2016a	May-16	PaPaS	PR/PGIC	AE, AEW, SAE, Sleep etc	4	296	2	155
Quetiapine	Seidel 2013	Jan-13	PaPaS	PI/PR	Satisfaction/QoL	12	772	0	0
**Cannabinoids**									
Nabilone	Walitt 2016b	Apr-16	PaPaS	PR/PGIC	AE, AEW, SAE, Sleep etc	2	72	0	0
**Opioids**									
Oxycodone	Gaskell 2016a	Jul-16	PaPaS	PR/ PGIC	W and AE	0	0	0	0
**Other**									
NSAIDs:<br>etoricoxib 90 mg daily<br>ibuprofen 2400 mg daily<br>naproxen 1000 mg daily<br>tenoxicam 20 mg	Derry 2017	Jan-17	PaPaS	PR/PGIC	AE, AEW, SAE, Sleep etc	6	292	2	146
Combinations of analgesics:<br>amitriptyline with fluoxetine (89 participants; 1 study)<br>amitriptyline with a different agent (92 participants; 2 studies)<br>melatonin with an antidepressant (164 participants, 2 studies)<br>carisoprodol, paracetamol (acetaminophen), and	Thorpe 2018	Sep-17	PaPaS	PR/ PGIC	W and AE	14	1289	no meta-analysis	

						Number included		Number included in the main pain analysis	
Drug	Author, year	Date of last search	Review Group	Main primary Pain measures	Other measures	Trials	Participants	Trials	Participants
caffeine (58 participants; 1 study)<br>tramadol and paracetamol (acetaminophen) (315 participants, 1 study)<br>malic acid and magnesium (24 participants; 1 study)<br>a monoamine oxidase inhibitor with 5-hydroxytryptophan (200 participants; 1 study)<br>pregabalin with duloxetine (41 participants; 1 study).									

MSG = Musculoskeletal group; NMD = Cochrane Neuromuscular Disease group; PaPaS = Cochrane Pain, Palliative and Supportive Care Review Group; PR = pain relief; PGIC = Patient Global Impression of Change; W = withdrawal; AE = adverse events; AEW = adverse event withdrawal; SAE = serious adverse events; QoL = quality of life

Supplementary table S8: AMSTAR-2 scores for the included studies (1 if present, 0 if absent, N/A is not applicable, usually as no trial data)

		AMSTAR 2 Questions																	
		1	2	3	4	5	6	7	8	9	10	11	12	13	14	15	16		
Intervention	Author, year	PICO	Protocol established before review	Study design explanation	Comprehensive literature search	Study selection in duplicate	Data extraction in duplicate	List of exclusions + justification	Included studies in detail	RoB assessment satisfactory	Sources of funding for studies	Appropriate meta/analysis	Impact of RoB on result	Impactof RoB in discussion or interpretation	Heterogeneity investigated discussed	Publication bias (Small study bias)	CoI of review authors reported	Other comments	AMSTAR confidence
Amitriptyline	Moore 2015	1	1	0	2	1	1	1	1	1	0	1	1	1	1	1	1	2 non critical	Moderate
Antipsychotic	Walitt 2016a	1	1	0	2	1	1	1	1	1	1	1	1	1	1	1	1	1 non critical	High
Cannabis	Walitt 2016b	0	1	0	2	1	1	1	1	1	1	N/A	N/A	N/A	1	N/A	1	Limited assessment 2 non critical	Moderate
Carbamazepine	Wiffen 2014	1	1	0	2	1	1	1	1	1	0	1	1	1	1	1	1	1 non critical	High
Clonazepam	Corrigan 2012	1	1	0	2	1	1	1	1	1	N/A	N/A	N/A	1	N/A	N/A	1	Limited assessment 1 non critical	Moderate
Combination	Thorpe 2018	1	1	0	2	1	1	1	1	1	0	N/A	N/A	N/A	1	N/A	1	Limited assessment 2 non critical	Moderate
Duloxetine	Lunn 2014	1	1	0	2	1	1	1	1	1	0	1	1	1	1	1	1	2 non critical	Moderate
Gabapentin	Cooper 2017	1	1	0	2	1	1	1	1	1	0	AMSTAR	N/A	1	1	N/A	1	Limited assessment 2 non critical	Moderate
Lacosamide	Hearn 2012	1	1	0	2	1	1	1	1	1	0	1	1	1	1	1	1	2 non critical	Moderate
Lamotrigine	Wiffen 2013b	1	1	0	2	1	1	1	1	1	0	1	1	1	1	1	1	2 non critical	Moderate
MAOI	Tort 2012	0	1	0	2	1	1	1	1	1	1	1	1	1	1	1	1	2 non critical	Moderate
Milnacipran	Cording 2015	1	1	0	2	1	1	1	1	1	0	1	1	1	1	1	1	2 non critical	Moderate
Mirtazepine	Welsch 2018	1	1	0	2	1	1	1	1	1	1	1	1	1	1	1	1	1 non critical	High
NSAID	Derry 2017	1	1	0	2	1	1	1	1	1	1	1	1	1	1	1	1	1 non critical	High
Oxycodone	Gaskell 2016	1	1	0	2	1	1	1	1	1	N/A	1	N/A	N/A	N/A	N/A	1	Limited assessment 1 non critical	Moderate
Phenytoin	Birse 2012	1	1	0	2	1	1	1	1	1	N/A	1	N/A	N/A	N/A	N/A	1	Limited assessment 1 non critical	Moderate
Pregabalin	Derry 2016	1	1	0	2	1	1	1	1	1	1	1	1	1	1	1	1	1 non critical	High
SNRI	Welsch 2018	1	1	0	2	1	1	1	1	1	1	1	1	1	1	1	1	1 non critical	High
SSRI	Walitt 2015	0	1	0	2	1	1	1	1	1	1	1	1	1	1	1	1	2 non critical	Moderate
Topiramate	Wiffen 2013c	1	1	0	2	1	1	1	1	1	0	1	N/A	1	1	1	1	2 non critical	Moderate
Valproate	Gill 2011	1	1	0	2	1	1	1	1	1	0	1	N/A	1	1	N/A	1	Limited assessment 2 non critical	Moderate

Notes: Three reviews did not define a full PICO. Only eight of 14 reviews that found any trials gave funding sources for included trials (there were six reviews with no trials that could not report on funding sources). None of the included reviews provided a statement regarding the appropriateness of the designs of studies to be examined. All 21 reviews had a previously published protocol, mentioned study selection and data extraction being carried out by two people, satisfactorily described exclusions and provided details of inclusion criteria, had an adequate assessment of the risk of bias, and included statements about any conflicts of interest. Most reviews scored a partial "yes" for searching because they did not search any grey literature, but we judged this adequate for identifying efficacy assessment studies for pain; two reviews received full scores.

Supplementary table S9: Scoring the critical pain criteria (1 if present, 0 if absent)

Intervention	Author, year	Critical pain criteria<br>17<br>Defined diagnostic criterion	18<br>Patient reported pain only	19<br>Defined minimum pain intensity	20<br>Study size: sensitivity analysis for small studies	21<br>Susceptibility to publication bias	22<br>Missing data - LOCF/Not mentioned or BOCF	23<br>Studies properly randomised	24<br>Studies properly double blind	Sum CPC
Amitriptyline	Moore 2015	1	1	1	1	1	1	1	1	8
Antipsychotic	Walitt 2016a	1	1	1	1	1	1	1	0	7
Cannabis	Walitt 2016b	1	1	1	1	1	1	1	1	8
Carbamazepine	Wiffen 2014	0	1	1	1	0	1	1	1	6
Clonazepam	Corrigan 2012	0	1	0	0	0	0	1	1	3
Combination	Thorpe 2018	0	1	1	1	1	1	1	1	7
Duloxetine	Lunn 2014	0	1	1	0	1	0	1	1	5
Gabapentin	Cooper 2017	1	1	1	1	1	1	1	1	8
Lacosamide	Hearn 2012	1	1	1	1	1	1	1	1	8
Lamotrigine	Wiffen 2013b	0	1	0	0	0	1	1	1	4
MAOI	Tort 2012	1	0	0	0	0	0	1	1	3
Milnacipran	Cording 2015	1	1	1	1	1	1	1	1	8
Mirtazepine	Welsch 2018	1	1	1	1	1	1	1	1	8
NSAID	Derry 2017	1	1	1	1	1	1	1	1	8
Oxycodone	Gaskell 2016	1	1	0	1	1	1	1	1	7
Phenytoin	Birse 2012	0	1	0	0	0	1	1	1	4
Pregabalin	Derry 2016	1	1	1	1	1	1	1	1	8
SNRI	Welsch 2018	1	1	1	1	1	1	1	1	8
SSRI	Walitt 2015	1	0	1	0	1	1	1	1	6
Topiramate	Wiffen 2013c	0	1	1	1	1	1	1	1	7
Valproate	Gill 2011	0	1	1	0	1	1	1	1	6

Notes: Most reviews met the critical pain criteria, although judgement of individual criteria in individual reviews was hampered because reviews lacked specific statements about their requirements, and because a minority found few if any trials to include. In the absence of information, judgements had to be made about, for example, the minimum pain requirement based on details provided in the included studies. At least seven of the eight criteria were met by 13 of the 21 reviews, and only four reviews met fewer than five. Results for the critical pain criteria in individual reviews are in Supplementary file 9. The most consistently fully met questions were appropriate randomisation (21 reviews), blinding (20 reviews), and the requirement for patient reported pain (19 reviews). The most frequently missed questions were sensitivity to small study size (14 reviews), minimum pain intensity (16 reviews), and susceptibility to publication bias (16 reviews).

Supplementary table S10: Grade assessment by review and overview authors compared

Intervention	Author, year	GRADE reported for pain in original review	GRADE assessed by the overview review	Reason for difference
Amitriptyline	Moore 2015	Very low	Very low	
Antipsychotic	Walitt 2016a	Very low	Very low	
Cannabis	Walitt 2016b	Very low	Very low	
Carbamazepine	Wiffen 2014	Low or very low	Very low	No data rated as very low
Clonazepam	Corrigan 2012	No data for FMS	Very low	No data rated as very low
Combination	Thorpe 2018	Very low	Very low	
Duloxetine	Lunn 2014	Low	Moderate	Review downgraded due to indirectness and publication bias
Gabapentin	Cooper 2017a	Very low	Very low	
Lacosamide	Hearn 2012	No data for FMS	Very low	No data rated as very low
Lamotrigine	Wiffen 2013b	Not used for FMS	Very low	No data rated as very low
MAOI	Tort 2012			
Low	Very low	<200 participants for analysis		
Milnacipran	Cording 2015	Moderate or high	Moderate or high	
Mirtazepine	Welsch 2018b	Low or very low	Moderate or high	Downgrading due to exclusions criteria and susceptibility to PB
NSAID	Derry 2017	Very low	Very low	
Oxycodone	Gaskell 2016a	Very low	Very low	
Phenytoin	Birse 2012	No data for FMS	Very low	No data rated as very low
Pregabalin	Derry 2016	High	High	
SNRI	Welsch 2018a	Low	Moderate/high	Review downgraded due to indirectness and publication bias
SSRI	Walitt 2015	Very low	Very low	
Topiramate	Wiffen 2013c	No data for FMS	Very low	No data rated as very low
Valproate	Gill 2011	Not used	Very low	No data rated as very low

MAOI – monoamine oxidase inhibitors; NSAIDs – Non-steroidal anti-inflammatory drugs; PICO - population, intervention, comparison, outcome; SNRI - Serotonin and norepinephrine reuptake inhibitors; SSRI - Selective serotonin reuptake inhibitors

Supplementary data S11: Calculations for mirogabalin efficacy for pain relief

Data from: Arnold LM, Whitaker S, Hsu C, Jacobs D, Merante D. Efficacy and safety of mirogabalin for the treatment of fibromyalgia: results from three 13-week randomized, double-blind, placebo- and active-controlled, parallel-group studies and a 52-week open-label extension study. *Curr Med Res Opin* 2019;35: 825-35.

Three trials failed to meet the preset primary outcome for mirogabalin of **change in weekly average daily worst pain score at week 13**. A pooled analysis for numbers of participants with a PGIC score of 2 or below (much or very much improved) gave mean RD values of 0.14 (NNT 7; 95% CI 5.4 to 10) for pregabalin 300 mg daily, 0.09 (NNT 11; 95% CI 8 to 20) for mirogabalin 15 mg daily and 0.08 (NNT 13; 95% CI 8 to 27) for mirogabalin 30 mg daily. Data from the 1915 participants in the pregabalin 300 mg versus placebo comparison are substantial compared to the 1375 participants included in the Cochrane review of pregabalin. The Cochrane reviews calculated a RD of 0.09 (95% CI 0.04 to 0.14) and a NNT of 11 (95% CI 7.3 to 25) for a response of PGIC ≤2. Confidence in the existing Cochrane review of pregabalin would be increased by adding this substantial amount of data.
